# Advances in Inhaled Nanoparticle Drug Delivery for Pulmonary Disease Management

**DOI:** 10.1096/fj.202502624R

**Published:** 2025-11-10

**Authors:** Yunzhou Fan, Yuefang Zhou, Jing Zhao, Yutong Zhao

**Affiliations:** ^1^ Department of Physiology and Cell Biology, College of Medicine, Davis Heart and Lung Research Institute The Ohio State University Columbus Ohio USA

**Keywords:** COVID‐19, inhalation, intratracheal, nanoparticle, respiratory disease

## Abstract

Pulmonary disorders, notably highlighted by the global impact of COVID‐19, continue to pose serious public health concerns with limited treatment options. To address the urgent need for effective lung‐targeted therapies, strategies that maximize local therapeutic efficacy while minimizing systemic toxicity are essential to the unique anatomical location of the lungs, inhaled therapy provides a promising strategy for locally targeted drug delivery through intranasal or intratracheal administration. Integrating biomedical nanotechnology and inhaled therapy, inhaled nanoparticle drug delivery systems (INDDs) have emerged as a powerful platform capable of penetrating mucus and pulmonary surfactant barriers, enhancing lung distribution and retention, and precisely delivering small molecules, proteins, and nucleic acids in lung lesions and cells using natural or synthetic carriers. The INDDs provide a universal translational platform for structurally analogous drugs and a wide array of respiratory disorders. This review summarizes recent advances in INDDs, focusing on the critical carrier materials in formulation, performance in in vitro and in vivo, and inhalation dosage forms, highlighting design strategies to overcome lung barriers and improve clinical and preclinical therapeutic efficacy in lung diseases.

## Graphical abstract

Pulmonary disorders remain a global health challenge. This review explores critical innovations in inhaled nanoparticle drug delivery systems focusing on their potential to revolutionize treatment strategies for a wide spectrum of pulmonary diseases. We highlight recent breakthroughs in carrier materials, performance evaluations in vitro and in vivo, and emerging inhalation dosage forms, contributing meaningfully to ongoing research in respiratory therapeutics and nanomedicine.

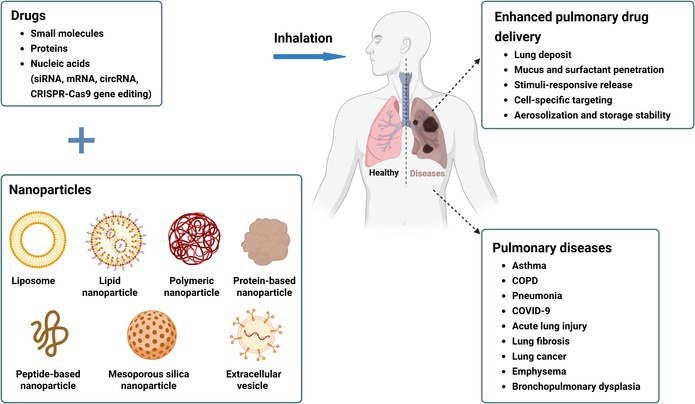

## 1 Introduction

Lung diseases, such as asthma, chronic obstructive pulmonary disease (COPD), pneumonia, lung cancer, COVID‐19, are a severe public health concern with a high morbidity and mortality, heavy healthcare and economic burden worldwide, and their therapy has urgent clinical need [1, 2]. As the unique physiological location of the lungs, non‐invasive inhaled medications can be directly delivered into the lungs through intranasal or intratracheal routes [3]. Compared to oral and intravenous administration, pulmonary drug delivery has the advantages of local lung targeting, high local drug concentration and rapid onset of action, low systemic distribution and toxicity, avoided degradation in the gastrointestinal tract and hepatic first‐pass metabolism, and good patient compliance without using needles [4, 5]. The clinical inhaled products, classified into nebulizer, dry powder inhaler, soft mist inhaler, and pressurized metered dose inhaler according to the inhalation devices, are commonly used in pulmonary delivery of small molecule drugs for asthma and COPD treatment [6].

The biomedical nanotechnology has been developed rapidly and is increasingly applied in inhaled medications. Inhaled nanoparticle drug delivery systems (INDDs) expand the range of drugs to insoluble small molecules, biomacromolecules such as proteins (e.g., antibodies, growth factors), nucleic acids (e.g., siRNA, mRNA, and the CRISPR‐Cas system), and enlarge disease indications to intractable pulmonary fibrosis, lung cancer, and respiratory viral infection [7, 8, 9, 10, 11, 12, 13, 14]. Compared with conventional inhaled systems, INDDs achieve the increase of drug solubility and bioavailability, penetration of mucus and pulmonary surfactant barriers, lung retention, active lung targeting, controlled or sustained drug release, avoided pulmonary clearance and lysosomal degradation, ultimately improving pulmonary drug delivery and therapeutic outcomes with reduced drug dose [8, 9, 10, 11, 12]. In 2018, FDA approved the first INDDs, Arikayce, amikacin liposome suspension for oral inhalation to treat *Mycobacterium avium* complex (MAC) lung disease. In the same year, lipid nanoparticles (LNPs) were first approved for the first siRNA drug Onpattro for intravenous injection to treat hereditary transthyretin (hATTR) amyloidosis. In 2021 and 2022, FDA approved two well‐known mRNA‐LNPs COVID‐19 vaccines, Comirnaty and Spikevax for intramuscular injection. These marketed products (Table 1) paved the way for the launch of new INDDs‐based drugs to treat lung diseases.

**TABLE 1:** Typical FDA‐approved nanoparticle‐based products.

Name	Active drug	Carrier and lipids components	Administration route	Indication	Company	Approval year
Doxil	Doxorubicin	Liposome: HSPC, Chol, MPEG‐DSPE (56:38:5, molar ratio)	Intravenous	Ovarian cancer	Sequus	1995
Arikayce	Amikacin	Liposome: DPPC, Chol (2:1, weight ratio)	Oral inhalation	MAC lung disease	Insmed	2018
Onpattro	Patisiran siRNA	LNPs: DLin‐MC3‐DMA, DSPC, Chol, PEG_2000_‐C‐DMG (50:10:38:1.5, molar ratio)	Intravenous	hATTR amyloidosis	Alnylam	2018
Comirnaty	mRNA^a^	LNPs: ALC‐0315, DSPC, Chol, ALC‐0159 (46.3:9.4:42.7:1.6, molar ratio)	Intramuscular	COVID‐19	Pfizer/BioNTech	2021
Spikevax	mRNA^a^	LNPs: SM‐102, DSPC, Chol, PEG_2000_‐DMG (50:10:38:1.5, molar ratio)	Intramuscular	COVID‐19	Moderna	2022
Abraxane	Paclitaxel	Albumin	Intravenous	Breast cancer	Abraxis	2005
Fyarro	Sirolimus	Albumin	Intravenous	PEComa	Aadi	2021

Abbreviations: ALC‐0159, 2‐(polyethylene glycol 2000)‐N,N‐ditetradecylacetamide; ALC‐0315, ((4‐hydroxybutyl)azanediyl)bis(hexane‐6,1‐diyl)bis(2‐hexyldecanoate); Chol, cholesterol; DLin‐MC3‐DMA, (6Z,9Z,28Z,31Z)‐heptatriaconta‐6,9,28,31‐tetraen‐19‐yl‐4‐(dimethylamino)butanoate; DPPC, dipalmitoylphosphatidylcholine; DSPC, 1,2‐distearoyl‐sn‐glycero‐3‐phosphocholine; HSPC, fully hydrogenated soy phosphatidylcholine; MPEG‐DSPE, N‐(carbonyl‐methoxypolyethylene glycol 2000)‐1,2‐distearoyl‐sn‐glycero‐3‐phosphoethanolamine sodium salt; PEG_2000_‐C‐DMG, α‐(3′‐{[1,2‐di(myristyloxy)propanoxy]carbonylamino}propyl)‐ω‐methoxy, polyoxyethylene; PEG_2000_‐DMG, PEGpolyethylene glycol 2000 dimyristoyl glycerol; SM‐102, 1‐Octylnonyl 8‐[(2‐hydroxyethyl)[6‐oxo‐6‐(undecyloxy)hexyl]amino]‐octanoate.

^a^ mRNA encoding spike glycoprotein of SARS‐CoV‐2.

With a deeper understanding of pathological mechanisms involved in lung diseases, the discovery of new targets and therapeutic agents, and progress in the development of INDDs, more medications are suitable for pulmonary delivery, offering improved treatment effectiveness and fewer side effects. In this review, we summarize the most recent advances on INDDs, including widely studied liposomes, LNPs, polymeric nanoparticles, protein‐ and peptide‐based nanoparticles, mesoporous silica nanoparticles, extracellular vesicles, and cell membrane‐derived nanovesicles, focusing on design strategies for enhancing mucus and surfactant penetration, targeting specific lung lesions and cells, and stability during inhalation and storage. We also emphasize both the potential and the challenges of INDDs in clinical application, offering insights into how these systems can serve as a flexible platform for drugs with similar structure and for treating a variety of lung diseases.

## 2 Mechanisms of Nanoparticle Deposition and Clearance in the Lungs

Inhaled particles can reach and deposit within the lungs through the respiratory tract, which is divided into upper (nose, nasal cavity, pharynx, and larynx), lower (trachea, bronchi, bronchioles) airways, and alveoli [15]. Mucus secreted by goblet cells lines the airways from nasal to bronchioles and traps inhaled particles [16]. Pulmonary surfactant, a complex mixture composed of lipids (e.g., DPPC) and proteins (e.g., SP‐A, SP‐B), is produced by alveolar type II (AT2) cells and lines the alveoli, which maintain lung function by lowering surface tension and also interact with inhaled particles [17]. Therefore, mucus and surfactant are crucial physiological barriers in the lungs, and certain respiratory diseases like COPD, asthma, and cystic fibrosis may cause mucus hypersecretion [18, 19]. In addition, extracellular matrix (ECM) and bacterial biofilm are pathological barriers in lung fibrosis and bacterial infection, respectively [20, 21]. Particles inhaled into the respiratory tract are subsequently removed through mucociliary clearance, the action of alveolar macrophages, and enzymatic breakdown [22].

**FIGURE 1:**
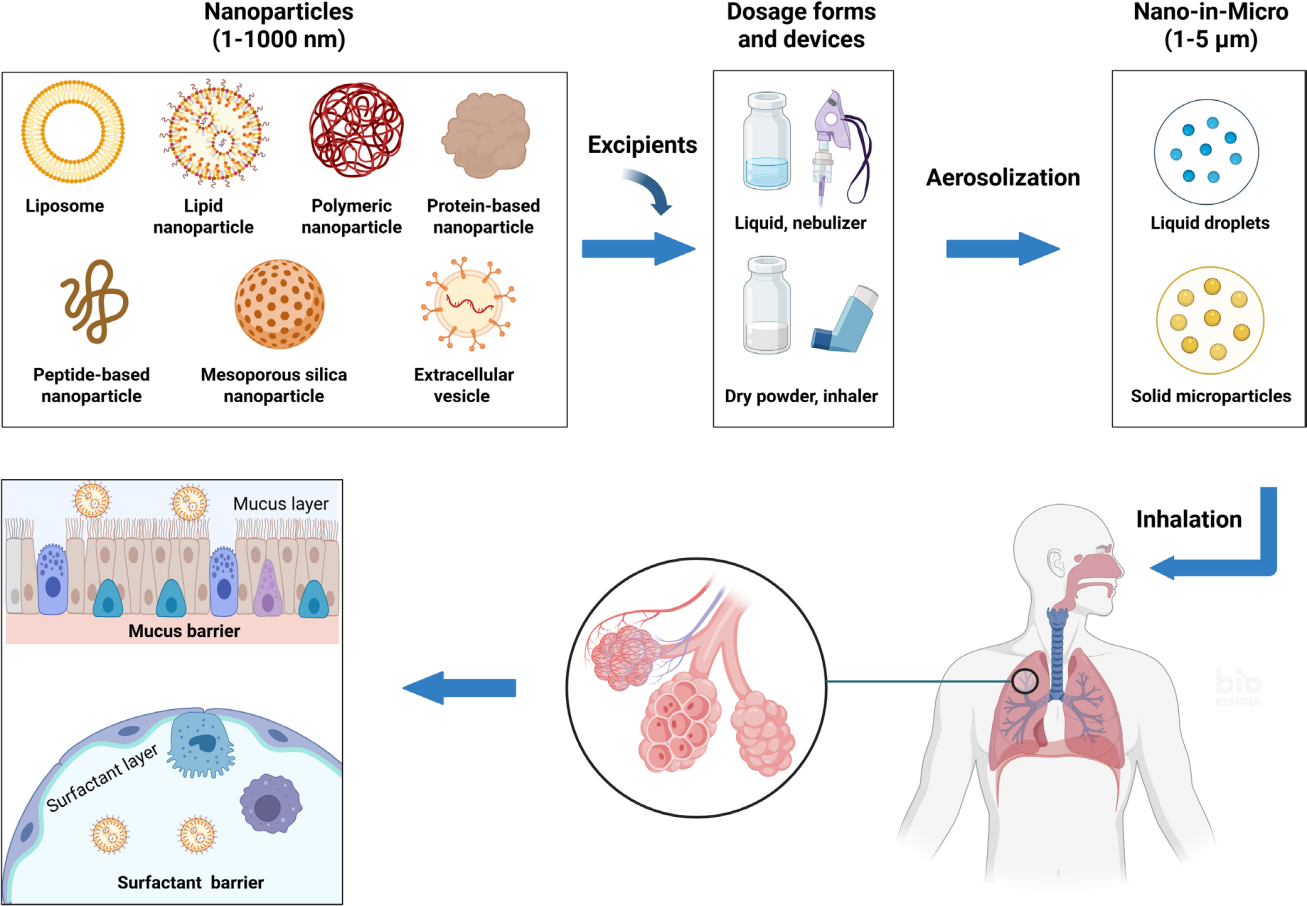
Inhaled nanoparticle drug delivery systems. The figure is created with BioRender.

As the airways progressively branch and narrow from the trachea to the alveoli, the aerodynamic size of inhaled aerosol particles is critical for their deposition site and extent in the lungs, primarily through mechanisms such as inertial impaction, gravitational sedimentation, and Brownian diffusion [23]. Particles larger than 5 μm typically settle in the upper airways due to inertial impaction; those between 1 and 5 μm tend to deposit in the lower airways via gravitational sedimentation; and particles smaller than 0.5 μm diffuse into the alveoli [22, 24, 25]. Other factors such as the physicochemical properties of the particles, airway anatomy, breathing pattern, and disease condition also affect the particles' deposition [26, 27]. It should be noted that inhaled nanoparticles are delivered in the form of micro‐sized aerosols during inhalation, either liquid droplets from nebulizers or solid microparticles from dry powder inhalers, rather than as individual nanoparticles [28, 29, 30, 31, 32]. Once deposited and exposed to lung fluids, inhaled aerosols dissolve and subsequently release their embedded nanoparticles [33]. The “Nano‐in‐Micro” strategy, which encapsulates nanoparticles within microparticles, enables efficient microparticle deposition in the lungs while facilitating the targeted delivery of nanoparticles, thereby harnessing their unique advantages as drug nanocarriers (Figure 1).

## 3 Targeted Delivery of Inhaled Nanoparticles to the Lung Lesions and Cells

Targeted delivery remains a significant challenge in pulmonary drug administration. A series of strategies has been developed to address the issue. Firstly, mucus‐ and surfactant‐penetrating nanoparticles can passively deposit and target the lung organ through controlling the aerodynamic size of aerosol particles [22, 34]. Secondly, inhaled nanoparticles can selectively target lung lesions and cells by employing intelligent stimuli‐responsive or active targeting strategies to further improve drug utilization and achieve more precise, efficient, and safe therapy for lung diseases without affecting normal lung tissue.

“Smart” stimuli‐responsive nanoparticles are engineered to release drugs triggered by physiological stimuli (e.g., low pH, hypoxia, high ROS, GSH, inflammation, and enzymes) within specific disease microenvironments, or exogenous energetic stimuli (e.g., temperature, light, ultrasound, and magnetic field). Upon exposure to these stimuli, the nanoparticles composed of responsive biomaterials undergo a change of structure and function, or degradation of components, consequently leading to localized or tailored drug release, activation of therapeutic agents, or cellular uptake. Tumor and pneumonia typically have an acidic microenvironment due to aberrant metabolism and lactic acid production [35, 36]. Inhalable pH‐responsive polymeric nanoparticles, DNA tetrahedron nanoparticles, mesoporous silica nanoparticles, albumin and peptide‐based nanoparticles are developed for treating lung cancer, pneumonia, and bacteria‐related COPD [36, 37, 38, 39, 40, 41, 42]. Tumor cells and inflamed cells have high ROS levels, causing high GSH levels to maintain redox homeostasis. Inhalable ROS‐ or GSH‐responsive liposomes, mesoporous silica nanoparticles, polymeric nanoparticles, protein‐ and peptide‐based nanoparticles are constructed for treating lung fibrosis, acute lung injury (ALI), pneumonia, and lung cancer [43, 44, 45, 46, 47, 48, 49]. Matrix metalloproteinases (MMPs), a family of enzymes that degrade ECM, are highly expressed in several lung diseases [50]. Inhalable MMP‐2‐ or MMP‐9‐responsive exosomes‐liposomes hybrid nanoparticles, nanoreactors, gelatin/silk fibroin composite microparticles, and nanogels are developed for treating ALI, lung cancer, lung fibrosis, and asthma [51, 52, 53, 54, 55]. These internal stimuli offer spatial control of medication, while external stimuli can achieve temporal control of medication. Inhalable ultrasound‐responsive metal–organic‐framework nanoparticles, magnetothermal and ultrasound‐responsive gadolinium‐doped iron sulfide nanoparticles are developed for treating pneumonia [56, 57].

Surface‐modified nanoparticles with targeting ligands such as peptides, antibodies, small molecules, and aptamers are tailored to actively recognize the pathological cells with unique receptors or biomarker proteins (Table 2) in lung diseases. Tumor cells highly express CD44, PD‐L1, integrins, glucose transporter, folate receptor, luteinizing hormone‐releasing hormone (LHRH) receptor, and transferrin receptor to support their rapid growth and survival. Inhalable nanoparticles conjugated with hyaluronic acid, anti‐PD‐L1 antibody, RGD peptide, mannose, folic acid, LHRH peptide, and transferrin are developed to target tumor cells and show enhanced therapeutic efficiency in lung cancer [60, 61, 66, 69, 70, 71]. Activated macrophages overexpress CD44, glucose transporter, CD206, and folate receptor. Inhalable nanoparticles decorated with hyaluronic acid, mannose, dextran, anti‐CD206 antibody, and folic acid are constructed to target macrophages for treating lung cancer and pneumonia [11, 61, 64]. The aberrant activation and transformation of fibroblast to myofibroblast is one key mechanism of lung fibrosis. Fibroblast activation protein (FAP), fibronectin, CD44, and integrins are overexpressed in these cells. Inhalable nanoparticles conjugated with FAP peptide, fibronectin peptide, hyaluronic acid, anti‐CD44 antibody, and RGD peptide are developed to target lung fibroblast or myofibroblast for treating lung fibrosis [10, 59, 62, 63].

**TABLE 2:** Active targeting strategies for inhaled nanoparticles.

Targeting receptors or proteins	Targeted cells	Diseases	Ligands	References
Neonatal Fc receptor (FcRn)	Epithelium and macrophages	Asthma	FcRn peptide	[58]
Fibronectin	Myofibroblasts	Lung fibrosis	CREKA peptide	[59]
Fibroblast activation protein (FAP)	Myofibroblasts	Lung fibrosis	FAP peptide	[10]
Integrins	Myofibroblasts and injured AT2 cells	Lung fibrosis	RGD peptide	[14]
	Cancer cells	Lung cancer	RGD peptide	[60]
CD44	Cancer cells	Lung cancer	Hyaluronic acid	[61]
	Macrophages	Pneumonia	Hyaluronic acid	[61]
	Fibroblasts	Lung fibrosis	Hyaluronic acid or anti‐CD44 antibody	[62, 63]
Glucose transporter	Cancer cells	Lung cancer	Mannose	[61]
	Macrophages	Pneumonia	Mannose	[61]
CD206	Tumor associated macrophages Macrophages	Lung cancer Pneumonia	Dextran or anti‐CD206 antibody Mannose	[11, 64] [65]
Folate receptor	Cancer cells	Lung cancer	Folic acid	[66]
	Macrophages	Pneumonia	Folic acid	[67]
LHRH receptor	Cancer cells	Lung cancer	LHRH peptide	[68]
Transferrin receptor (TfR)	Cancer cells	Lung cancer	Transferrin or TfR peptide	[69, 70]
PD‐L1	Cancer cells	Lung cancer	Anti‐PD‐L1 antibody	[71]

## 4 Classification of INDDs and Therapeutic Efficacy in Lung Diseases

Nanoparticles typically have a size of 1–1000 nm [72, 73]. The widely used INDDs include liposomes, LNPs, polymeric nanoparticles, protein‐ and peptide‐based nanoparticles, mesoporous silica nanoparticles, biomimetic nanoparticles such as extracellular vesicles and cell membrane‐derived nanovesicles, which have demonstrated therapeutic effects in lung diseases via loading small molecules, proteins, and nucleic acids (Table 3).

**TABLE 3:** Representative applications of inhaled synthetic nanoparticles for lung disease treatment.

INDDs	Drugs	Diseases	Targeted cells	Pharmacological effects
Liposomes	Pachypodol	ALI	Macrophages	Anti‐inflammation and repairing lung epithelial‐endothelial barriers [8]
	Nintedanib/ABT‐263	Lung fibrosis	Aged epithelial cells, fibroblasts	Removing senescent epithelial cells and inhibiting fibroblasts activation [74]
	Verteporfin/pirfenidone	Lung fibrosis	Epithelial cells, fibroblasts	Inhibiting honeycomb cyst formation and fibroblast overactivation [75]
	ASSNAC/nintedanib	Lung fibrosis	Fibroblasts	Inhibiting inflammation caused by nintednib [76]
	Nintedanib/IL11 siRNA	Lung fibrosis	Myofibroblasts	Inhibiting fibroblast activation and profibrotic immune response [10]
	Osimertinib/GF2BP3 siRNA	Lung cancer	Tumor cells	Inhibiting EGFR in lung tumor and GF2BP3 in brain tumor [77]
	Erdafitinib	Lung cancer	Premetastatic niche (PMN)	Regressing pulmonary PMNs [78]
	CRISPR/Cas9 targeting HK2	ALI	Macrophages	Reprogramming macrophage metabolism and induced inflammation [79]
LNPs	mCOVID, mOVA, mGP70^a^	COVID‐19, lung cancer	DCs	Inducing robust systemic and mucosal immune responses [12]
	IL‐11 scFv mRNA	Lung fibrosis	Immune and mesenchymal cells	Inhibiting fibroblast activation and ECM production [80]
	CYB5R3/BMP4 mRNAs	Lung fibrosis	AT2 cells	Promoting realveolarization via restoring AT2 stemness [81]
	Budesonide/mnbTSLP^b^	Asthma	Immune and epithelial cells	Reducing inflammation and restoring steroid resistance [82]
	Budesonide/HGF mRNA	Emphysema	Epithelial cells	Inhibiting inflammation and alveoli wall thinning [83]
	Ursolic acid/NR1D1 mRNA	BPD and lung fibrosis	Epithelial and immune cells	Reducing inflammation, ROS, lung fibrosis, and lung injury [84]
	mCOVID^a^	COVID‐19	APCs	Induction of mucosal immunity [85]
	Antigen‐coding circRNA	Lung Cancer	APCs	Eliciting T cell response against lung cancers [86]
Polymeric nanoparticles	Tobramycin	Pneumonia	Bacteria	Eliminating lung bacteria and inflammation [39]
	DNase I	Lung cancer	Tumor cells and NETs	Eliminating NETs and improving radiosensitization [87]
	FTY720/nobiletin	ALI	Macrophages, neutrophils	Inhibiting inflammatory responses [88]
	IL‐11 siRNA	Lung fibrosis	Epithelial, immune cells	Inhibiting fibroblasts activation, inflammation, ECM deposition [89]
	Indole acetic acid	COPD	Macrophages	Mitigating inflammation and augmenting pulmonary function [90]
	Tbx2 mRNA	Silicosis	Multiples cells	Inhibiting ROS and inflammation [91]
	Platinum prodrug	Lung cancer	Tumor cells	Inhibiting GST activity, GSH level, releasing NO [92]
	poly(I:C)	Lung cancer	APCs	Inducing potent antitumor immunity [93]
	Dexamethasone	Asthma	Immune cells	Inhibiting inflammation and airway epithelium thickening [58]
	Ciprofloxacin, vancomycin	Pneumonia	Bacteria	Improving anti‐bacterial effects by enhanced distribution, retention [32, 94]
	Doxorubicin	Lung cancer	Tumor cells	Improving anti‐tumor effects by enhanced distribution, retention [95]
Protein‐ and Peptide‐based nanoparticles	MMP13 mRNA, KGF	Lung fibrosis	Myofibroblasts, injured AT2 cells	Boosting ECM clearance and alveolar re‐epithelialization [14]
	Cerium‐based tannic acid	Pneumonia	Macrophages, epithelial cells	Reducing ROS, inflammation, viral load and lung damage [46]
	Peptides	Pneumonia	Macrophages	Anti‐inflammation, bacterial eradication, promoting alveoli regeneration [96]
	Curcumin	Lung cancer	Tumor cells	Enhancing curcumin uptake and release in tumor cells [47]
	Polymyxin B	Pneumonia	Bacteria	Reducing bacterial load and inflammatory response [38]
	RBD antigen	COVID‐19	APCs	Inducing strong production of IgG and IgA, local T cell response [97]
MSNs	Doxorubicin, AMP	Lung cancer	Tumor cells, bacteria	Simultaneously killing commensal bacteria and tumor cells [48]
	Iron	Lung cancer	TAMs, CSCs	Enhancing iron metabolism in TAMs to induce CSCs ferroptosis [11]
	Ceftazidime	COPD	Bacteria	Eradicating bacteria, promoting inflammation resolution [40]

^a^ mCOVID, mOVA, mGP70: mRNA encoding spike protein of SARS‐CoV‐2, ovalbumin, envelope glycoprotein 70.

^b^ mnbTSLP, mRNA encoding a thymic stromal lymphopoietin nanobody.

### 4.1 Liposomes

Liposomes are spherical, self‐assembled vesicles enclosed by a single lipid bilayer, primarily composed of amphiphilic phospholipids and cholesterol [98, 99]. The unique spatial arrangement structure of liposomes allows them to encapsulate hydrophilic and hydrophobic drugs in the inner aqueous core or lipid bilayer, thereby enhancing drug solubility, stability, and sustained release. Resembling the composition of mammalian cell membranes, liposomes have good biocompatibility and biodegradation. The additional flexibility of surface modification makes liposome a valuable and versatile drug carrier.

Doxil, doxorubicin hydrochloride liposome injection, is the first FDA‐approved nano‐drug for AIDS‐related Kaposi's sarcoma and ovarian cancer treatment, which is composed of HSPC, cholesterol, and DSPE‐PEG_2000_ [100, 101]. Arikayce is the first FDA‐approved inhaled liposome drug that is administered once daily by nebulization using the Lamira nebulizer system. Amikacin liposome comprises DPPC and cholesterol with a size of 200–300 nm [102]. The successful launch of Arikayce paves the way for further exploration of inhaled liposomes and other nanoparticles in clinical applications.

As an endogenous phospholipid and a main component of surfactant, DPPC has a high surfactant‐penetrating, lung‐targeting capability and excellent biocompatibility [103]. PEG‐lipids, like DSPE‐PEG_2000_, are frequently used to enhance the mucus penetration of nanoparticles [76, 104]. Pachypodol and bergapten, two natural compounds with anti‐inflammatory properties, have limited clinical application due to poor solubility. Sun et al. and Liao et al. developed inhalable pachypodol and bergapten liposomes, respectively, using DPPC, cholesterol, and DSPE‐PEG_2000_ [7, 8]. After nebulization, both liposomes target the inflamed lung and ameliorate ALI in mice via promoting anti‐inflammatory M2 macrophage polarization.

Nintedanib and pirfenidone are two FDA‐approved drugs for the treatment of idiopathic pulmonary fibrosis (IPF) through the inhibition of fibroblast activation and ECM production. While both drugs only delay IPF progression, they do not reverse the disease or significantly improve patient survival [105], suggesting multiple mechanisms are involved and targeting them with combination therapies may have the potential to improve the anti‐fibrotic effects. Yang et al. developed inhalable liposomes (soy lecithin, cholesterol, DSPE‐PEG_2000_‐NH_2_) co‐loaded with nintedanib and ABT‐263, featuring dual mucus‐ and ECM‐penetrating capabilities achieved via surface modification with tris‐(2‐carboxyethyl)‐phosphine and L‐arginine [74]. Following nebulization, the liposomes uniformly distribute in all lung lobes, released ABT‐263 selectively eliminates senescent epithelial cells, and suppresses the secretion of profibrotic factors that activate myofibroblasts, thereby synergistically improving the therapeutic efficacy of nintedanib in treating IPF. Han et al. developed inhalable liposomes (PC, cholesterol, DSPE‐PEG_2000_) co‐loaded with verteporfin and pirfenidone. Following intratracheal atomization, verteporfin reduces the fluidization of airway epithelium to alveoli and the formation of honeycomb cysts, while pirfenidone reduces fibroblast overactivation, jointly reversing IPF and restoring lung function [75]. Zhang et al. developed inhalable S‐allylmercapto‐N‐acetylcysteine (ASSNAC) and nintedanib co‐loaded liposomes (phospholipid, cholesterol, DSPE‐PEG_2000_) [76]. ASSNAC, with an anti‐inflammatory property, inhibits nintedanib‐induced inflammation. After nebulization, the co‐loaded liposomes at a 30‐time lower dose of nintedanib show superior anti‐fibrotic efficacy in rats compared to the oral nintedanib product. Chen et al. developed inhalable myofibroblast targeting liposomes (DOTAP, cholesterol) co‐loading nintedanib and IL‐11 siRNA [10]. After intratracheal inhalation, the liposomes superiorly deposit in the lung and selectively target myofibroblasts. Released nintedanib and IL‐11 siRNA synergistically inhibit fibroblast activation and ECM deposition, promote epithelium repair, and remodel the immune microenvironment, notably improving pulmonary fibrosis.

Lung cancer is a prevalent and deadly disease that can metastasize to other organs, while other cancers can also spread to the lungs [106, 107]. Fu et al. developed inhalable osimertinib and DNA plasmid encoding IGF2BP3 siRNA co‐loaded liposomes (lecithins, cholesterol), which are coated with a stem cell membrane enriched with surfactant SP‐B [77]. Following nebulization, the liposomes are largely retained in the lungs with the help of SP‐B, where they target tumor cells and release their therapeutic payload. Osimertinib inhibits oncogenic EGFR signaling in tumors, while DNA plasmid‐derived siRNA downregulates IGF2BP3 expression and induces the production of rabies virus glycoprotein‐engineered exosomes carrying IGF2BP3 siRNA. These exosomes further target brain tumors, resulting in effective suppression of both primary lung cancer and metastatic brain tumors. Wang et al. developed inhalable erdafitinib‐loaded liposomes (soybean phospholipid, cholesterol, DSPE‐PEG‐Maleimide) with surface modification of CXCL12‐binding W4 peptide [78]. After nebulization, the liposomes capture and accumulate in the CXCL12 highly expressed pulmonary pre‐metastatic niche (PMN), resulting in the release of erdafitinib to mitigate fibrosis, PMN formation, and lung metastasis in a mouse model of breast cancer. Notably, triple therapy combined with liposomal inhalation, CXCR4 antagonist spray hydrogel in the resection cavity of breast tumor, and Doxil chemotherapy significantly prolongs postoperative mouse survival.

Liposomes are also employed for pulmonary mRNA delivery. Hexokinase 2 (HK2) is a critical enzyme involved in glucose metabolism [108]. Huang et al. developed inhalable liposomes (DOPS, cholesterol, DSPE‐PEG_2000_) loading a complex core of calcium phosphate‐mRNA based CRISPR/Cas9 system targeting HK2. After nebulization, the liposomes induce HK2 knockout in alveolar macrophages and extensively inhibit lung inflammation and ALI in mice [79]. To address the nebulization instability and incompatibility with the pulmonary microenvironment (e.g., pulmonary surfactant and low serum) of mRNA‐LNPs, Jang et al. developed inhalable ionizable mRNA‐loaded liposomes (Dlin‐MC3‐DMA, cholesterol, DSPC) [109]. After nebulization, the liposomes widely distribute in the deep lung and target epithelial cells, which have therapeutic potential for various pulmonary diseases.

### 4.2 LNPs

LNPs have emerged as a key nonviral carrier for RNA delivery. LNPs are formed by the self‐assembly of four‐component lipids including ionizable lipid, helper phospholipid, cholesterol, and PEG‐lipid [110]. DLin‐MC3‐DMA, ALC‐0315, and SM‐102 LNPs are clinically exploited as carriers to deliver siRNA and mRNA (Table 1) [111]. mRNA‐based therapeutics that encode proteins, vaccine antigens, and gene editors hold tremendous promise for treating a wide range of diseases [112]. Comirnaty and Spikevax have shown efficacy and safety as mRNA‐LNPs vaccines with intramuscular injection [113], paving the avenue for the application of inhaled mRNA‐LNPs in lung disease treatment. From 2023, several inhaled mRNA‐LNPs drugs, including ARCT‐032, VX‐522, RCT1100, and RCT2100 have started clinical trials for treating cystic fibrosis or primary ciliary dyskinesia [114].

The formulation of inhaled mRNA‐LNPs is primarily based on the benchmark formulations of marketed LNPs. Maintaining stability during nebulization remains a significant challenge. The high shear forces of nebulization can potentially disrupt LNPs' integrity and cause LNPs disintegration, aggregation, and mRNA leakage, leading to unsatisfactory delivery and transfection efficiency. For example, only 17% SM102 LNPs remain intact after nebulization [12]. Inhaled LNPs formulations are optimized to improve their stability and effectiveness during aerosolization by adjusting lipids' ratios, using novel ionizable lipids (e.g., IR‐117‐17, IR‐19‐Py), and zwitterionic polymer‐functionalized lipid as a replacement of PEG‐lipid [80, 115, 116]. Liu et al. developed inhalable charged‐assisted stabilization (CAS) of LNPs by integrating peptide‐conjugated DOPE into SM102 LNPs [12]. CAS‐LNPs exhibit exceptional stability during nebulization and efficiently deliver mRNA into the lungs of mice, dogs, and pigswhile primarily targeting dendritic cells (DCs). Following nebulization, CAS‐LNPs elicit robust systemic and mucosal immune responses, demonstrating therapeutic efficacy as both a COVID‐19 vaccine and a cancer vaccine.

In addition to FDA‐approved ionizable lipids, other novel ionizable lipids with superior performance are developed for pulmonary mRNA delivery. As IL‐11 is a potential target for treating IPF, Bai et al. optimized inhalable AA3‐Dlin LNPs to load mRNA encoding IL‐11 single chain fragment variable (scFv) [80]. Following nebulization, the LNPs effectively deliver IL‐11 scFv mRNA into the lungs and produce antibody to neutralize IL‐11, significantly ameliorating lung fibrosis through suppression of fibroblast activation and ECM deposition. Cytochrome b5 reductase 3 (CYB5R3) and bone morphogenetic protein 4 (BMP4), two essential genes for alveolar epithelial repair, are downregulated in AT2 cells during IPF. Wang et al. developed inhalable mucus‐penetrating GAE14 LNPs encapsulating dual mRNAs of CYB5R3 and BMP4 [81]. After nebulization, expressed CYB5R3 reduces mitochondrial damage and attenuates AT2 cell senescence, while BMP4 alleviates impaired AT2‐mediated epithelial remodeling and suppresses fibroblast activation. Together, these effects contribute to the dual mitigation of lung fibrosis and improved survival in mice. Thymic stromal lymphopoietin (TSLP) released from injured airway epithelial cells contributes to steroid resistance in severe asthma [117]. Tezepelumab, a clinical human monoclonal antibody binding to TSLP, is used to treat severe asthma via subcutaneous injection [118]. Huang et al. developed inhalable AA3‐Dlin LNPs co‐loading TSLP mRNA and budesonide [82]. After nebulization, the LNPs distribute in the whole lung and primarily target immune and epithelial cells. Produced TSLP antibody blocks the upstream immune cascade and restores steroid resistance, while budesonide suppresses the transcription of downstream inflammatory genes, jointly alleviating asthma symptoms in severe asthma mice and steroid‐resistant asthmatic mice, compared to inhaled budesonide product or injected tezepelumab. CD44 and glucose transporters are highly expressed in cancer cells and macrophages [119, 120, 121, 122]. Tang et al. developed polymer‐lipid hybrid mRNA‐LNPs, composed of cationic lipid G0‐C14, polymer hyaluronic acid targeting CD44, and DSPE‐PEG‐Mannose targeting glucose transporters [61]. After inhalation, the LNPs exhibit excellent dual‐targeting ability and effectively deliver mRNA into cancer cells in a murine model of orthotopic lung cancer, as well as proinflammatory macrophages in a murine model of pneumonia.

In addition, biodegradable ionizable lipids are designed to build LNPs with lower toxicity and more efficacy. Li et al. synthesized a new biodegradable ionizable lipid RCB‐4‐8 to develop inhalable LNPs, which efficiently delivered mRNA or CRISPR‐Cas9 mRNA for genome editing into the mice airway epithelium [123]. Huang et al. developed inhalable LNPs using degradable ionizable glycerolipid TG4C to load mRNA encoding hepatocyte growth factor (HGF) [83]. The LNPs show high stability, uniform distribution in the lungs, and primarily target epithelial cells. After intratracheal administration, HGF mRNA‐LNPs significantly attenuate lung inflammation and alveoli wall thinning, and ameliorate pulmonary emphysema in mice. To address the immunogenicity and suboptimal pulmonary mRNA delivery of LNPs, Zhao et al. constructed a biodegradable, cationic phosphoramide‐derived lipid PL32 and developed non‐inflammatory PL32 LNPs by incorporating ursolic acid, which enhanced mRNA expression and relieved inflammation via activating vacuolar‐type ATPase (V‐ATPase) and consequently promoting endosome acidification [84]. The LNPs mainly target epithelial and immune cells in the lungs and enhance lung protein expression by 40 folds without causing inflammation compared to ALC‐0315 LNPs. After nebulization, LNPs loading mRNA encoding the nuclear receptor subfamily 1 group D member 1 (NR1D1), a circadian regulatory gene, markedly improve efficiency in treating bronchopulmonary dysplasia (BPD) in rats and pulmonary fibrosis in mice.

Intranasal delivery is also utilized for treating respiratory system diseases. Maniyamgama et al. designed liquid lipid nanoparticles (iLLNs) with a liquid lipid core for intranasal mRNA delivery, using lipid components of ALC‐0315, DOTMA, ‐sitosterol, DOPE, triolein, and DSPE‐PEG [85]. The near‐electroneutral, muco‐inert surface, and high deformability of iLLNs render them a good muco‐penetrating ability to cross the nasal epithelium. mRNA‐iLLNs predominantly induce protein expression in the noses and lungs. Intranasal immunization of mRNA‐iLLNs encoding spike protein of SARS‐CoV‐2 notably elicits mucosal immunity at the upper airway, showing prophylactic potential for COVID‐19 infection and transmission. In addition to mRNA, LNPs are also used to deliver circular RNA (circRNA). Li et al. developed intranasal circRNA vaccine using SM102 LNPs [86]. After intranasal administration, circRNA‐LNPs initiate protein expression in the lungs, and induce antigen‐specific T cells immune responses via targeting alveolar macrophages and DCs, leading to robust anti‐tumor efficacy against lung cancer. Moreover, the intranasal circRNA vaccine has combined effects with CAR‐T cell therapy.

### 4.3 Polymeric Nanoparticles

Polymeric nanoparticles are gaining increasing attention for pulmonary drug delivery, as their polymeric structure can be conjugated with specific groups to enable efficient delivery of therapeutic agents [124, 125]. Commonly used polymers include both natural (e.g., starch, chitosan, alginate) and synthetic types (e.g., PLGA, poly(lactic‐co‐glycolic acid); PEG; PEI, polyethylenimine). PLGA has been approved by the FDA for clinical application with excellent biocompatibility, biodegradation, and sustained‐release properties [126].

Starch is a natural neutral polysaccharide with high biocompatibility. Ren et al. developed inhalable tobramycin (TOB)‐loaded oxidized soluble starch nanoparticles modified with mPEG‐NH_2_ [39]. Following nebulization, the nanoparticles effectively penetrate the mucus and release TOB in the acidic environment of the bacteria‐infected site, and ameliorate bacterial pneumonia and inflammation. ROS generation during radiation induces the formation of neutrophil extracellular traps (NETs), compromising the anti‐tumor effects of radiation [127]. Sun et al. developed inhalable DNase I‐loaded PLGA nanoparticles coated with gold (Au) nanoparticles [87]. After nebulization, Au nanoparticles enhance radiation sensitization, while DNase I eliminates NETs formation. Together, the dual‐action effectively inhibits lung metastasis in mice. Both as anti‐inflammatory agents, FTY720 exhibits frequent adverse effects, while nobiletin has poor solubility [128, 129]. Zhang et al. developed inhaled FTY720 and nobiletin co‐loaded PLGA nanoparticles [88]. After nebulization, the nanoparticles distribute in lung alveoli and notably target macrophages and neutrophils, suppress cytokine release, immune cell infiltration, and NF‐κB expression in the lungs, enhancing lung recovery in ALI mice. As IL‐11 is a pivotal cytokine involved in IPF, Dong et al. developed inhalable IL‐11‐siRNA nanoparticles using PEI modified with 4‐guanidinobenzoic acid [89]. Following nebulization, the nanoparticles are accumulated in lung fibrotic lesions and primarily target epithelial, endothelial, and immune cells, ultimately attenuating IPF progression and improving lung functions.

New polymeric materials are being developed and applied in pulmonary drug delivery. Indole acetic acid (IAA), a microbial metabolite with anti‐inflammatory properties, has poor solubility limiting its application. Wang et al. developed IAA‐loaded generation 4 polyamidoamine nanoparticles [90]. After nebulization, the nanoparticles mainly retain in the lungs, target, and promote macrophage polarization toward the anti‐inflammatory M2 phenotype, reducing inflammation and improving lung function in COPD mice. T‐box transcription factor 2 (Tbx2), a crucial transcription factor in lung development and growth, is down‐regulated in silicosis. Yong et al. synthesized polyplexes 20% b‐3C‐2P12, a “four‐in‐one” LNPs‐like highly branched polys (β‐amino ester) by integrating the four components of LNPs to deliver mRNA encoding Tbx2 [91]. After nebulization, Tbx2 mRNA‐loaded nanoparticles effectively restore lung function in silicosis mice. Glutathione S‐transferase (GST)‐induced conjugation of GSH with cisplatin contributes to cisplatin resistance in cancer [130]. Tang et al. developed inhalable PEOz‐b‐PLA‐GSNO nanoparticles modified with targeting molecule dibenzyl cyclooctyne (DBCO) to deliver a platinum prodrug composed of ethacrynic acid (EA, a GST inhibitor) and cisplatin [92]. With targeting and entering into bioorthogonal molecule azide‐prelabeled tumor cells, the nanoparticles release EA under the acidic and high GSH environment to inhibit GST activity, while released nitric oxide (NO) from donor GSNO triggered by GSH improves cisplatin uptake, both processes pronouncedly deplete GSH due to simultaneous GSH consumption, collectively reversing cisplatin resistance and inhibiting the growth of cisplatin‐resistant orthotopic non‐small cell lung cancer after nebulization.

Polymeric nanoparticles can integrate with other types of nanoparticles to create hybrid nanocarriers. Inspired by the virus structure, Li et al. developed inhalable anionic polymer hyaluronic acid‐bisphosphonate‐based nanogel within LNPs to deliver an immunostimulatory agent poly(I:C) [93]. After intratracheal inhalation, the nanoparticles predominantly distribute in lung tumors and target DCs and macrophages, robustly activating antitumor immunity and suppressing lung metastases. Furthermore, poly(I:C) and PD‐L1 siRNA co‐loaded nanoparticles exhibit a synergistic therapeutic effect. Neonatal Fc receptor (FcRn) is overexpressed in lung epithelium and macrophages. Yu et al. developed inhalable dexamethasone‐loaded PLGA‐lipid nanoparticles with surface modification of FcRn peptide, which facilitates transepithelial transport of nanoparticles and subsequent entry into inflammatory cells [58]. After intranasal administration, the nanoparticles uniformly distribute and retain in the lungs, reduce inflammation and airway epithelium thickness, effectively reversing asthma in mice. The Wang group developed cell membrane‐coated PLGA nanoparticles loading drugs, followed by conjugation with natural algae to create a series of biohybrid microrobots [32, 94, 95]. Integrating the active motility of algae, cell‐mimicking properties of cell membranes (e.g., neutrophil, red blood cell, platelet), and sustained‐release capability of PLGA nanoparticles, the dynamic microrobots uniformly distribute into deep lungs, prolong lung retention, and evade clearance by alveolar macrophages. After intratracheal administration or nebulization, ciprofloxacin‐, doxorubicin‐, and vancomycin‐loaded microrobots effectively inhibit acute *Pseudomonas aeruginosa* pneumonia, lung metastasis, and acute methicillin‐resistant *Staphylococcus aureus* pneumonia, respectively.

### 4.4 Protein‐ and Peptide‐Based Nanoparticles

Protein and peptide utilize amino acids as fundamental building blocks. Protein‐based nanoparticles can be prepared using emulsion, self‐assembly, and desolvation methods, while peptide‐based nanoparticles are spontaneously self‐assembled peptide nanostructures driven by the forces of intermolecular interactions [131, 132, 133]. Both nanoparticles possess excellent properties of stability, biocompatibility, biodegradation, and low immunogenicity. In 2005 and 2021, the FDA approved Abraxane, paclitaxel albumin protein‐bound nanoparticles (~130 nm) for intravenous use to treat breast cancer, and Fyarro, sirolimus albumin protein‐bound nanoparticles for intravenous use to treat perivascular epithelioid cell tumor (PEComa), respectively. Non‐bioactive peptide nanoparticles can act as drug carriers, while certain bioactive peptide nanoparticles can also be utilized as therapeutics or for enhanced targeting and cell penetration.

Polymyxin B (PMB) is recognized as the last‐line antibiotic against multidrug‐resistant Gram‐negative bacteria; however, it has severe neurotoxicity and nephrotoxicity when administered by intravenous injection. Li et al. developed inhaled pH‐responsive PMB‐loaded albumin nanoparticles conjugated with an acid‐responsive molecule (PEBA) and Amine‐PEG‐Thiol [38]. The thiol groups with a mucolytic effect, mucus‐inert PEG modification, and negative charge of nanoparticles enable them to achieve good mucus penetration. After aerosol inhalation, the nanoparticles distribute in the alveoli and release PMB in the acidic microenvironment of the bacterial infection area, decreasing lung bacterial load and inflammation in pneumonia mice infected with carbapenem‐resistant *Klebsiella pneumonia*. Zhang et al. constructed inhalable ribosomal protein‐based and RGD peptide‐modified nanoparticles co‐loading MMP13 mRNA and keratinocyte growth factor (KGF) [14]. The nanoparticles release KGF to decrease intrapulmonary ECM in fibrotic lesions, while MMP13 mRNA is delivered in integrin‐enriched myofibroblasts and injured AT2 cells to re‐epithelialize the disrupted alveolar epithelium, synergistically improving lung function and reversing pulmonary fibrosis in mice. Notably, the inhaled nanoparticles show combined effects with oral pirfenidone and provide a potential strategy for IPF treatment. Jeong et al. developed inhalable thiolated mussel adhesive protein‐based nanoparticles loading curcumin [47]. Following nebulization, the nanoparticles retain in the lungs and target tumor cells through thiol‐mediated internalization, then release curcumin triggered by high levels of GSH in tumors, resulting in significantly suppressing lung metastasis in mice.

Compared to proteins, peptides exhibit a simpler structure, less immunogenicity, and superior design flexibility in the modification of peptide sequences and conjugation with various molecules to tailor their properties and functions [134, 135]. Peng et al. developed inhalable self‐assembling peptide‐based nanoparticles conjugated with cerium‐based tannic acid nanozyme [46]. The peptide self‐assembles and aggregates into β‐sheets under high ROS levels in inflamed areas, driving the nanozyme to form an active fibrous structure displaying enzyme‐like antioxidant properties. Following nasal inhalation, the nanozyme primarily targets the macrophages and epithelial cells, and effectively alleviates lung inflammation, lung damage, and viral load through neutralization in a murine model of viral pneumonia. Chen et al. constructed inhalable self‐assembly peptide‐based nanoparticles conjugated with a membrane‐permeable motif [96]. Following intratracheal injection, the nanoparticles accumulate in injured lungs and specifically target macrophages, resulting in inhibiting proinflammatory responses, expediting lung regeneration through promoting M2 macrophage polarization, and eradicating bacteria through membrane disruption, collectively extending the survival rate in bacteria‐induced ALI mice. To solve the low efficiency and cytotoxicity during the intracellular delivery of nucleic acids and proteins, Eweje et al. constructed a recombinant elastin‐like polypeptide fused to an endosomal escape peptide, and developed self‐assembling nanoparticles [9]. The nanoparticles superiorly deliver siRNA, mRNA, plasmid DNA, proteins, and CRISPR gene editors to multiple cell lines and primary cells in vitro. Furthermore, intranasal instillation of the nanoparticles loading Cre recombinase protein effectively delivers protein to lung epithelial cells and induces gene editing in mice, while the benchmark SM‐102 LNPs show minimal effects. Pentameric cholera toxin B subunit (CTB) is a potent mucosal adjuvant. Ye et al. constructed self‐adjuvanting self‐assembled nanoparticles by fusion of a trimer‐forming peptide to CTB, displaying the SARS‐CoV‐2 receptor‐binding domain (RBD) antigen [97]. The nanoparticles are incorporated into porous PLGA microcapsules to form a dry powder with a suitable aerodynamic size. After inhalation, the microcapsules predominantly deposit in the alveoli, and released nanoparticles mainly target the antigen‐presenting cells (APCs) and induce robust systemic and mucosal immune responses, effectively protecting against SARS‐CoV‐2 in mice, hamsters, and nonhuman primates. Furthermore, inhaled vaccine co‐displaying wild‐type RBD and Omicron variant RBD significantly prevents Omicron transmission in hamsters.

### 4.5 Mesoporous Silica Nanoparticles

Mesoporous silica nanoparticles (MSNs) are inorganic nanoparticles characterized by a porous structure that enables the encapsulation of therapeutic agents. The structural features of MSNs, such as high surface area, adjustable size and volume, and ease of surface modification, make them promising drug carriers. These properties support high drug loading, controlled release, targeted delivery, as well as good biocompatibility and biodegradability [136, 137].

Bacterial infections are a significant factor contributing to poor survival outcomes in patients with lung cancer [138]. Ma et al. developed inhalable MSNs co‐loading doxorubicin (DOX) and antimicrobial peptide (AMP) [48]. Following nebulization, MSNs biodegradation is caused by high GSH in the tumor microenvironment, followed by the release of DOX and AMP to kill tumor cells and commensal bacteria simultaneously, achieving an improved therapeutic effect against lung cancer in tumor/bacterial commensal mice. Tumor‐associated macrophages (TAMs) provide a high level of iron required for cancer stem cells (CSCs) growth, while excessive iron may induce CSCs ferroptosis [139, 140]. Feng et al. developed nebulized iron‐doped MSNs [11]. After nebulization, MSNs are taken up by TAMs through endocytosis and degraded in the lysosome. This process releases elevated levels of iron and reduces the antioxidative glucose‐6‐phosphate within lung tumor microlesions, collaboratively enhancing CSCs ferroptosis and suppressing tumor progression in an early orthotopic lung cancer model. We should note that in this study iron‐based nanoparticles regulate TAMs and indirectly trigger CSCs ferroptosis. It is worthwhile to explore new strategies that directly target and induce CSCs ferroptosis in future research. The hypersecretion of mucus and formation of bacterial biofilm prevent effective antimicrobials delivery for COPD treatment [141]. Zhu et al. developed inhalable ceftazidime (CAZ)‐loaded MSNs gated with polypeptides [40]. These nanoparticles effectively penetrate mucus and biofilm, and polypeptides are activated under the acidic environment in biofilm to disrupt bacterial membranes, scavenge bacterial DNA, and unmask MSNs pores to release CAZ. The combined antibacterial, antioxidative, and anti‐inflammatory effects of inhaled nanoparticles synergistically facilitate the resolution of inflammation and improve lung function in patients with COPD.

Nevertheless, occupational exposure to crystalline silica dust in industrial settings may lead to silicosis, characterized by lung inflammation and fibrosis [142]. The inhalation of MSNs raises the potential safety concerns regarding their possible role in inducing pulmonary fibrosis. MSNs, a form of amorphous silica, possess a distinct biosafety profile compared to crystalline silica. Recent studies have shown that synthetic amorphous silica does not induce lung fibrosis in rats [143, 144, 145].

### 4.6 Biomimetic Nano‐Delivery Systems

Biomimetic nano‐delivery systems represent a novel biological approach to drug delivery, offering key advantages such as high biocompatibility, targeted delivery, and prolonged circulation or retention [146]. Recently, inhaled extracellular vesicles and cell membrane‐derived nanovesicles have been widely employed in the treatment of lung diseases.

#### 4.6.1 Extracellular Vesicles

Extracellular vehicles (EVs) are lipid bilayer‐bound particles secreted by cells that carry proteins, lipids, nucleic acids, and other biomolecules [147]. They play pivotal roles in intercellular communication, antioxidative defense, immune modulation, and processes of tissue repair and regeneration [148, 149]. EVs, including exosomes, are being actively investigated as therapeutic agents and drug delivery systems for transporting nucleic acids, proteins, and small‐molecular drugs. Moreover, EVs can be bioengineered to display specific targeting ligands, antigens, or therapeutic molecules, enhancing their precision and effectiveness in disease treatment.

Several nebulized EVs have undergone clinical trials, demonstrating both therapeutic efficacy and favorable safety profiles in the treatment of lung diseases. Examples include adipose tissue mesenchymal stromal cell (MSC)‐derived exosomes, convalescent human immune plasma‐derived exosomes, umbilical cord mesenchymal stem cell‐derived exosomes, and exosomes expressing CD24 used for treatment of COVID‐19 and related acute respiratory distress syndrome [150, 151, 152, 153, 154, 155]. Additionally, human umbilical cord MSC‐derived EVs have demonstrated their safety and efficacy in treating pulmonary fibrosis [156]. Extensive preclinical studies also suggest the therapeutic potential of inhalable EVs for treatment of lung diseases. For example, mesenchymal stem cell‐derived exosomes for COPD; platelet‐derived EVs for emphysema; human embryonic kidney cell‐derived exosomes loading IL‐12 mRNA; CAR‐T cell‐derived exosomes loading paclitaxel for lung cancer; hypoxic human umbilical cord MSC‐derived EVs (Hypo‐EVs), or Hypo‐EVs loading miR‐146a‐5p for asthma; lung‐derived exosomes loading mRNA encoding spike protein, or conjugated with RBD of SARS‐CoV‐2 for COVID‐19; EVs expressing club cell protein, and exosomes co‐loading RAGE‐binding peptide and curcumin for ALI [157, 158, 159, 160, 161, 162, 163, 164, 165].

Limited yield and inefficient drug‐loading present significant challenges to the application of EVs. As an alternative to exosomes, nebulized milk‐derived exosomes carrying TGF‐β1 siRNA significantly ameliorate pulmonary fibrosis and improve mouse survival rate [166]. Fusion of EVs with liposomes can combine the advantages of both carriers, leveraging the natural targeting capability of EVs and the high‐loading capacity of liposomes. Liu et al. developed inhalable serum exosomal and liposomal hybrid nanoparticles conjugated with DNase I to load methylprednisolone sodium succinate (MPS) [51]. The nanoparticles effectively distribute in the lungs and penetrate mucus by DNase I conjugation. In inflamed alveoli, elevated MMP‐9 expression triggers the release of DNase I, accelerating NETs degradation, while MPS directs macrophages toward M2 polarization, collectively enhancing therapeutic efficacy in an ALI murine model. Wang et al. developed inhalable chitosan microparticles encapsulating cryptotanshinone (CTS)‐loaded liposome‐exosome hybrid vesicles [59]. Leveraging the fibronectin‐binding specificity of CREKA peptide‐modified liposomes and the homing capabilities of exosomes, the hybrid vesicles effectively facilitate CTS delivery into lung myofibroblasts, thereby enhancing the anti‐fibrotic efficacy in a rat model.

#### 4.6.2 Cell Membrane‐Derived Nanovesicles

Cell membrane‐derived nanovesicles (NVs) are nano‐sized vesicles prepared from extracted cell membranes, which are utilized as an alternative drug delivery system to EVs due to the limitation of EV production [167, 168]. By preserving the membrane proteins and lipids, NVs retain the distinct characteristics and functional capabilities of their parental cells.

Similar to EVs, mesenchymal stem cell‐derived NVs administered intranasally alleviate allergic airway inflammation in mice [169]. Meng et al. developed inhalable neutrophil‐derived NVs engineered with cholesterol to deliver dexamethasone (DEX), named as nanoDEX [170]. By preserving the surface chemokine and cytokine receptors, nebulized nanoDEX largely retains in inflamed lungs, and targets macrophages and DCs, mitigates COVID‐19 cytokine storm through down‐regulation of cytokine production by DEX and cytokine neutralization by nanovesicles, synergistically attenuating lung inflammation and injury in mice and rhesus macaques. Meanwhile, nanoDEX effectively suppresses DEX‐induced osteoporosis in rats. Yu et al. developed inhalable neutrophil membrane‐coated PLGA nanoparticles loading levofloxacin (LVX) [171]. With mimicking neutrophil, the nanoparticles with intratracheal injection actively penetrate the mucus and arrive at the inflammatory sites, escape macrophage clearance and prolong the retention in the lungs, eliminate the bacteria by LVX and neutralize cytokines by the neutrophil membrane, achieving therapeutic efficiency in bacteria‐infected COPD. MicroRNA155 (miR155) is aberrantly expressed during ALI; Zhuang et al. developed inhalable lung epithelial cell membrane‐derived NVs loading anti‐miR155 oligonucleotide, effectively ameliorating LPS‐induced ALI in mice [172].

In addition, genetically engineered cells expressing specific proteins or antigens can be used to produce NVs with targeting or antigen delivery capabilities. Wang et al. developed inhalable nanovaccines by fusion of RBD‐overexpressed 293 T cell‐derived NVs with monophosphoryl lipid A (MPLA)‐loaded liposomes, possessing antigen presentation, adjuvant, and surfactant‐penetrating properties [173]. After nebulization, the nanovaccines effectively deliver RBD into alveolar macrophages and activate macrophages by MPLA, induce robust mucosal and systemic immunity, protecting against SARS‐CoV‐2 pseudovirus infection in mice. Zhu et al. developed inhalable anti‐PD‐L1 scFv expressing HEK293T cell‐derived nanovesicles loading STING agonist 2′3′‐cGAMP [71]. After nebulization, the nanovesicles accumulate in the lungs, target and deliver 2′3′‐cGAMP to high PD‐L1‐expressed tumor cells, activate STING‐mediated immunostimulatory tumor microenvironments, enhancing the antitumor effects of CAR‐T cells. Furthermore, the nanovesicles block PD‐L1 on the tumor and prevent CAR‐T cell exhaustion, leading to combined effects with CAR‐T cell therapy in inhibiting tumor growth in orthotopic lung cancer and lung metastasis mouse models.

## 5 Dosage Forms of INDDs

Nebulizers and dry powder inhalers are the most utilized inhalation dosage forms for inhaled nanoparticles. Formulating nanoparticles suitable for inhalation presents considerable challenges. Firstly, desirable aerodynamic properties of generated aerosols, specifically a mass median aerodynamic diameter (MMAD) of 1–5 μm and a high fine particle fraction (FPF, the fraction of the emitted dose with aerodynamic diameter < 5 μm), are required for effective lung deposition. MMAD and FPF, determined by the Next Generation Impactor or aerodynamic particle counter, allow for an evaluation and selection of the formulation and inhalation devices [47, 174]. Secondly, good aerosolization and storage stability are crucial for effective pulmonary drug delivery, requiring consistent morphology, size distribution, zeta potential, encapsulation efficiency, and transfection efficiency before and after aerosolization, and before and after storage.

### 5.1 Nebulizer

For nanoparticle formulation in liquid solution or suspensions, favorable MMAD and FPF of aerosolized liquid droplets can be achieved by choosing appropriate inhalation devices (e.g., jet nebulizers, vibrating mesh nebulizers) [175]. For Arikayce, the MMAD of nebulized aerosol droplets is about 4.7 μm, and the FPF of the aerosol ranges from 50.3% to 53.5% [174]. The MMAD and FPF of ASSNAC and nintedanib co‐loaded liposomes are 1.85 μm and 81.1%, respectively [76]. The MMAD and FPF of thiolated mussel adhesive protein nanoparticles loading curcumin are 4.4 μm and 53%, respectively [47]. During nebulization of Arikayce, a portion of amikacin is released from liposomal encapsulation, resulting in the pulmonary delivery of both free and liposome‐associated amikacin. However, proteins and nucleic acids released from inhaled nanoparticles are susceptible to enzymatic degradation, compromising their transfection and delivery efficacy [12]. To enhance their stability during nebulization, formulation components such as buffering agents (e.g., sodium acetate, HEPEs), low salt buffers (e.g., 0.1 × PBS), and polymeric excipients (e.g., branched PEG20K, poloxamer 188) are incorporated [12, 80, 116]. Both mRNA COVID‐19 vaccines from Moderna and Pfizer/BioNTech use sucrose as a cryoprotectant for storage at frozen conditions [176]. Sucrose effectively enhances the post‐nebulization stability of mRNA‐LNPs at −20°C for 30 days, while LNPs without sucrose exhibit stability at 4°C for 7 days and instability at −20°C for 30 days [82]. Zhao et al. prepared lyophilized powder of mRNA‐LNPs with sucrose by freeze drying, which maintained protein expression in the lungs after reconstitution with water and stability at 4°C for over 90 days [84].

### 5.2 Dry Powder Inhaler

For dry powder‐based nanoparticle formulations, the MMAD and FPF of solid microparticles are predominantly influenced by the formulation composition and drying technology employed to encapsulate the nanoparticles. Dry powder inhalers have advantages of ease of use, portability, good drug stability, and accurate delivery dose [177].

Trehalose is commonly used as a matrix‐forming excipient in dry powder formulations for pulmonary delivery of nanoparticles. Inhalable dry powders of siRNA‐loaded lipid‐polymer hybrid nanoparticles with trehalose, and miR‐335‐loaded EVs with trehalose and leucine are prepared by spray drying and thin‐film freeze‐drying, respectively, both of which exhibit excellent aerosolization properties (MMAD: 2.96 μm, FPF: 65%; MMAD: 1.2 μm, FPF: 75.7%) while preserving the biological function of siRNA and miRNA [178, 179]. Dry powders of mRNA‐LNPs with trehalose and trileucine are prepared by spray drying, which show better stability and mRNA functionality stored at room temperature compared to liquid formulations stored at 4°C for 2 weeks [180]. Moreover, nanoparticles can be loaded in microparticulate drug delivery systems. Inhalable chitosan microparticles encapsulating CTS‐loaded liposomes and liposome‐exosome hybrid vesicles show good aerosolization performance (MMAD: 2.4 μm, FPF: 54%) and stability with storage under accelerated conditions for 3 months [59]. Inhalable clofazimine‐loaded MSNs exhibit excellent aerodynamic properties (MMAD: 1.65 μm, FPF: 50%) and stability for 12 months in refrigerated conditions [181]. Notably, the MSNs dissolve in lung fluid to release nanoparticles, thereby establishing a dual micro‐nano delivery platform.

## 6 Conclusions and Perspectives Challenges

The lungs, as essential respiratory organs, are vulnerable to a broad spectrum of diseases. INDDs offer substantial therapeutic potential by improving targeted drug delivery and bioavailability within pulmonary tissues. Clinical and preclinical investigations have shown that inhaled nanoparticle‐based therapeutics can achieve effective treatment outcomes with reduced drug doses or dosing frequency, and through combination strategies that co‐deliver multiple therapeutic agents with different targets. Among nanoparticle‐based therapeutics, the demonstrated safety and efficacy of recently approved inhaled liposomes and intramuscularly administered mRNA‐LNPs for treating lung diseases are expected to accelerate the clinical translation of inhaled liposomal formulations for other drugs, as well as the development of mRNA‐LNPs for pulmonary delivery.

Despite promising advances, inhaled nanoparticle formulations continue to face significant hurdles, including penetration through mucus and pulmonary surfactant barriers, maintaining aerosolization and storage stability. Formulation of inhaled nanoparticles should be designed and optimized to address these obstacles. Immunogenicity and biosafety raise further concerns regarding inhaled nanoparticles, potentially leading to lung inflammation, lung injury, and systemic toxicity. Biomimetic nanoparticles typically demonstrate high biocompatibility, low immunogenicity, and enhanced biosafety. The size, surface charge, composition, shape, stiffness, and hydrophobicity of inhaled nanoparticles can be fine‐tuned to avoid immunogenicity and toxicity [182, 183]. The immunogenicity of LNPs is effectively ameliorated by incorporating dexamethasone as an anti‐inflammatory agent, ursolic acid as a V‐ATPase agonist, budesonide as a partial replacement of cholesterol, thiodigalactoside or olitigaltin as galectin inhibitors, and using 4A3‐SC8 as a unique ionizable lipid limiting endosomal damage [82, 84, 184], and these strategies can be extrapolated to other inhaled nanoparticles. It is also essential to evaluate the minimum toxic dose and long‐term safety of repeated inhalation. Moreover, rodents such as mice and rats, mostly used in preclinical studies, possess respiratory structures and disease condition models that differ from those of humans, resulting in discrepancies in in vivo drug distribution and pharmacological responses. Large animals including dogs, pigs, and monkeys are increasingly used to evaluate the distribution, lung protein expression, and immune responses of inhaled nanoparticles, improving the clinical relevance and translational potential [12, 84, 97, 160].

Drugs within the same class often share similar structures and physicochemical properties. For example, different mRNAs encoding different proteins remain a consistent fundamental structure. A successful inhaled nanoparticle formulation will be versatile and adaptable to other drugs or other lung diseases. Besides the introduced INNDs in section 4, other types of INDDs, such as nanoemulsions, DNA origami nanostructures, metal–organic frameworks, and topologically engineered supramolecular cyclolipid nanoparticles, have been reported for treating lung diseases [185, 186, 187, 188, 189]. Recent advancements in inhaled liposomal and mRNA‐LNPs technologies highlight their promise for expanding therapeutic options in the treatment of pulmonary disorders. In a forward‐looking perspective, the innovative evolution in INDDs is focusing on integrating artificial intelligence (AI), developing “smart” stimuli‐responsive and cell‐specific targeting behaviors, and enabling personalized medicine.

Firstly, AI has guided the design of LNPs with two novel ionizable lipids, including FO‐32 and FO‐35, screened from a million lipids in silico, which show better pulmonary mRNA delivery than previously reported lipids [190]. In addition to in vivo delivery, the adaptability of AI will drive optimization of inhaled nanoparticles with superior performance in lung targeting, immunogenicity, safety, aerosolization, and storage stability. Moreover, NCK‐interacting kinase (TNIK) as a first‐in‐class target for IPF and rentosertib as a first‐in‐class TNIK inhibitor are discovered using AI [191, 192]. Rentosertib shows promising efficacy and safety in patients with IPF in clinical trials.

Secondly, stimuli‐responsive and active targeting nanoparticles using multifunctional biomaterials improve therapeutic outcomes and reduce adverse effects. These nanoparticles achieve on‐demand drug release in a programmed and spatiotemporal manner at the lung lesions and cells. Inhaled nanoparticles with cell‐specific delivery are primarily based on the interaction between targeting ligands on nanoparticles and specific receptors or proteins on targeted cells. It is critical to understand the role of diverse cell types in various lung diseases, identify the targeted cell types and specific cell surface biomarkers, and select high‐affinity ligands for nanoparticle targeting. For example, pulmonary delivery of nanoparticles with different ligands shows competitive uptake by cancer cells and tumor‐associated macrophages, resulting in different anti‐tumor efficacy in orthotopic lung tumors [66]. Further studies are needed for a better understanding of the nano‐bio interactions, targeting mechanisms, and validation in clinical trials.

Thirdly, inhaled nanoparticles facilitate personalized medicine. Genomics techniques identifying individual genetic mutations and specific disease biomarkers guide personalized medicine using gene therapy, protein, and small‐molecule. Personalized neoantigen vaccines have shown efficacy in treating lung cancer [193, 194]. Inhaled nanoparticles provide state‐of‐the‐art carriers for personalized medicine. Furthermore, theranostic nanoparticles integrating diagnostic imaging and therapeutic functions allow for real‐time monitoring of treatment response and guide personalized medicine by timely treatment adjustment [195, 196]. Collectively, these advances in inhaled nanoparticles will ultimately enable effective management of pulmonary diseases.

## Author Contributions

Y.F. and Y.Z. searched research articles and drafted the manuscript. J.Z. edited the manuscript. Y.Z. supervised the project and finalized manuscript.

## Conflicts of Interest

The authors declare no conflicts of interest.

## Data Availability

The authors have nothing to report.
